# Epidemiology and Genetics of Rheumatic Diseases Suggest a Constant Rate of DNA Damage as Underlying Cause

**DOI:** 10.1111/imm.70077

**Published:** 2025-12-07

**Authors:** Piet C. de Groen

**Affiliations:** ^1^ Division of Gastroenterology, Hepatology & Nutrition University of Minnesota Minneapolis Minnesota USA

**Keywords:** constant DNA damage, ionising radiation, MHC/HLA, monogenic diseases, muons, mutation, rheumatic diseases, somatic hypermutation hotspots, Standard Model, two‐hit hypothesis

## Abstract

The cause of rheumatic diseases is poorly understood; many appear to have a dominant inheritance with low, incomplete penetrance. A recent theory poses that all DNA is continuously damaged at a constant rate, causing a constant rate of mutations. Here, the hypothesis is tested that a constant, low rate of somatic mutations explains the low, incomplete penetrance of autoimmune rheumatic diseases and the increased penetrance of monogenic inflammatory rheumatic disease driven by multiple DNA loci prone to somatic mutation. Monogenic rheumatic diseases are proposed to require two mutations according to the two‐hit hypothesis by Knudson: a germline mutation on one allele, and a somatic mutation initiating rheumatic disease on the wild‐type allele. Two approaches are taken. The first one investigates whether the epidemiology of autoimmune rheumatic diseases adheres to two expected characteristics: a linear prevalence of disease and a tapering distribution of multiple disease events in individuals at risk. The second approach analyses at‐risk DNA for evidence of hypermutable loci: somatic hypermutation (SHM) hotspots. Autoimmune and monogenic inflammatory rheumatic diseases provide an opportunity to determine whether more than one similar SHM hotspot leads to earlier onset of disease and a higher degree of disease penetrance. Results show that examples of rheumatic diseases, such as rheumatoid arthritis, systemic sclerosis, lupus and Sjogren's syndrome, show a linear prevalence and an exponential distribution of one or more additional autoimmune diseases. SHM hotspots in HLA and non‐HLA genes in at‐risk people are associated with the risk of rheumatic diseases, and the difference in the number of SHM hotspots, one in autoimmune and PLB1 arthritis and several in COPA syndrome, associated with autoimmune and monogenic non‐HLA rheumatic diseases, explains the time of onset of disease and the degree of incomplete penetrance. This clarifies why autoimmune rheumatic diseases are inherited as true autosomal dominant traits with incomplete penetrance and non‐HLA monogenic rheumatic diseases as pseudo‐dominant traits with incomplete penetrance in accordance with the two‐hit hypothesis. Therefore, epidemiology and genetics are compatible with a constant rate of DNA damage and associated somatic mutations as the cause of autoimmune and monogenic rheumatic diseases among at‐risk people.

## Graphical abstract

A constant rate of DNA damage that is not perfectly repaired will cause a constant rate of DNA mutations. The chance of mutation will increase if DNA is prone to damage, such as occurs in somatic hypermutation (SHM) hotspots and GC‐rich DNA. Thus, if one mutation‐prone DNA site drives disease, the age of onset of disease and degree of penetrance should be similar (shown for HLA‐DRB1‐driven rheumatoid arthritis and single‐locus‐driven monogenic PLB1 arthritis); however, if multiple mutation‐prone DNA sites drive disease, early age of onset and high degree of penetrance can be expected (shown for multi‐locus‐driven COPA syndrome).

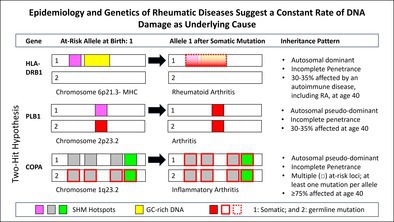

## 1 Background

The aetiology of rheumatic diseases is considered to be multifactorial, with a strong genetic component dominated by the MHC region in the genome. A recent theory posits that nearly all inherent human diseases are caused by somatic DNA mutations [1]. Moreover, the theory holds that most common autoimmune diseases are caused by somatic mutations within the critical regions of the MHC epitope‐binding groove important for discrimination of self‐ from non‐self‐proteins [2]. Finally, the theory states that most mutations arise through imperfect repair of DNA damage due to a constant flux of ionising radiation.

If the new theory is correct, and a single, critical mutation invokes a change of phenotype from normal to disease at a constant rate in the population, then the incidence of rheumatic diseases in an at‐risk population should be constant. Moreover, if the epitope‐binding groove somatic mutation concept is correct and a single mutation may cause a range of autoimmune diseases depending on the post‐mutation features of the epitope‐binding groove, then the distribution of additional autoimmune diseases (AAIDs) in people with autoimmune rheumatic diseases over a specific time of exposure to constant DNA damage should adhere to an exponential equation. For instance, among those at risk for autoimmune rheumatic disease, this may happen in 50% of people; that means that 25% have only a rheumatic disease, 12.5% have an AAID, 6.25% have two AAIDs, and the remaining 6.25% have three or more AAIDs during the time of observation. This concept was tested for type 1 diabetes (T1D); for all people with T1D, the number of AAIDs was determined, and as predicted, the distribution adhered to an exponential distribution [2]. The concept was also tested for a large number of malignancies that may occur more than once in a person's life [3]. Again, the distribution of additional cancers of the same type, and even different, unrelated types, tightly adhered to the predicted exponential distribution.

In addition to autoimmune diseases, there are monogenic rheumatic diseases. For some monogenic rheumatic diseases, the exact germline mutations are known; some are caused by a single mutation, and others can be caused by more than one mutation. Based on observations in the colon, where the APC gene needs to be inactivated on both alleles in a cell to cause polyp formation, the new theory proposes that inherited monogenic rheumatic diseases require a second mutation on the wild‐type allele in order to develop clinical disease. Thus, the new theory predicts that the onset of disease and the rate of penetrance depend on the number of at‐risk loci on the wild‐type allele.

Here, the two predictions derived from the new theory are tested to determine if the theory applies to rheumatic diseases. A wide range of rheumatic diseases was examined for a constant disease incidence or exponential distribution of associated AAIDs: ankylosing spondylitis, psoriatic arthritis, rheumatoid arthritis (RA), lupus, Sjogren's disease, systemic sclerosis and stiff‐person syndrome (SPS). The spectrum of AAIDs, the relationship among AAIDs, and the pros and cons of linkage studies versus genome‐wide association studies (GWAS) in rheumatic disease were investigated. Finally, the relationship between the number of at‐risk loci and both the age of disease onset and the degree of incomplete penetrance was evaluated. The results presented will show that predictions derived from the new theory are true: a constant rate of DNA damage can explain the cause of rheumatic diseases.

## 2 Methods

### 2.1 Disease Incidence and Distribution

The literature was surveyed for articles describing the type and frequency of rheumatic diseases that were associated with none, one or more than one AAID in the same person. Articles with detailed numeric information about the total population under study, the number of cases with rheumatic diseases and the type of autoimmune diseases were included; articles only describing categories of autoimmune diseases in percentages were excluded. For incidence studies, the number of cases per year was recorded. For distribution studies, the total number of cases, the number of cases with one or more autoimmune diseases, and the type of autoimmune diseases were recorded. When the number of individual autoimmune diseases and the total number of autoimmune diseases listed per person did not match (i.e., errors in the published article), or when only the total number of cases and AAIDs were listed, the cumulative number of autoimmune diseases and persons was used to estimate the number of autoimmune diseases per person.

The selection of articles was not random; the goal was to select articles that represented rheumatic diseases with differences in organ of origin, incidence, progression rate, histology, survival, geographic location, gender, race and time. The spectrum of AAIDs and any relationship among AAIDs was recorded, as were the pros and cons of linkage studies versus GWAS in rheumatic diseases. Monogenic diseases associated with rheumatic disease were selected as well.

The omnipresent neoplasia equations (ONE) derived from colonoscopy polyp studies as previously described were used to model the observed data [1, 4]. The versions of the ONE relevant to this study are:

(1) $$\mathrm{CE}\left(p\right)=X^{p}$$

where the chance that an event occurs *p* times is the event chance *X* to the power *p*

(2) $$f\left(p\right)=X^{p}/\left({Ʃ}_{p=0\to \infty }X^{p}\right)$$

where the fraction in the population with neoplastic diseases is for a specific value of *p*.

The constant *X* defines the event threshold at which a case develops a neoplastic lesion when belonging to the at‐risk group. As the chance of an event ranges from never to always, the possible values for *X* are 0 ≤ *X* ≤ 1. The equation was simplified based on empirical experience in endoscopy by setting a limit to the polyp number at ≥ 12. In rheumatic diseases, six or more autoimmune diseases in a single individual are rare events. Therefore, while keeping the same limit in the determination of the value of *X*, predictions were only displayed for *p* = 0–5. Although *X* can be determined based on two events (*p* = 2), any type of model (exponential, linear, etc.) will fit 2 points. To prevent this, observed data (i.e., autoimmune events) needed to consist of at least three observations and preferably four or more [3].

### 2.2 DNA Sequence Analysis

Germline DNA was analysed for hypermutable or mutation‐prone DNA sequences as previously described [2]. Somatic hypermutation (SHM) hotspots and GC‐rich sequences were identified using software programs and colour‐coded to allow rapid inspection of gene or CDS. Dominant inheritance with incomplete penetrance of non‐MHC genes was presumed to reflect a preexisting germline mutation on one allele, followed by somatic mutation of the second allele during life in the same cell; this is hypothesised to result in a biallelic loss of normal protein in the affected cell and an aberrant signal that is amplified to cause systemic disease. The concept mirrors the dominant inheritance with incomplete penetrance of the APC protein in people with familial adenomatosis coli (FAP). In the oncology literature, the concept is known as Knudson's hypothesis or two‐hit hypothesis [5]. A germline mutation on one allele with a somatic mutation on the second allele will cause polyposis or retinoblastoma when the APC and RB1 genes are affected, respectively. Most inherited mutations associated with FAP arise in SHM hotspots, and most colorectal tumours do not express functional APC protein compatible with a biallelic loss of wild‐type protein [6]. If many sites in the gene upon mutation are associated with the loss of function of the normal protein, then the age of onset is early and the lifetime tumour risk is very high. The DNA damage‐based theory thus applies the two‐hit concept to non‐cancer diseases, in particular, diseases with dominant inheritance with incomplete penetrance. The delayed incomplete penetrance reflects the time and likelihood of DNA damage and mutation of the not‐yet mutated wild‐type allele.

### 2.3 Statistics

The chance of rheumatic disease without or with one or more AAIDs shown in the example above adheres to an exponential decay distribution; if true for observed data, log_10_ transformation of observed data will result in a linear distribution that can be analysed using simple linear regression. The Pearson correlation measures the strength of the linear relationship between two variables [7]. In previous articles and preliminary studies, it was shown that the ONE predicted distribution of type I diabetes (T1D), neoplasia and pregnancy loss with near‐perfect accuracy; chi‐square goodness of fit tests and linear regression after log transformation confirmed that the model represented the observed data with great accuracy [1, 2, 3, 8]. Therefore, statistical analysis was limited to the Pearson correlation coefficient (PCC) and linear regression after log_10_ transformation for one dataset containing > 1 million persons with autoimmune diseases [7, 9]. The expected PCC for population analyses was ≥ 0.99 for studies with complete data and without confounding conditions [1]. A PCC > 0.9995 was rounded up to 1.

## 3 Results

The data analysed for this study covered a wide range of rheumatic diseases and consisted of information derived mostly from articles reporting on either retrospective chart reviews based on diagnosis registries, prospective local disease registries or prospective national disease registries. The diagnosis of initial and additional diagnoses was based on histology and serology.

Few studies met all selection criteria. Studies or databases were rejected when incidence and prevalence did not reflect stable populations due to population growth and inclusion over time of different ethnicities and minorities. Most articles reported odds ratios for additional diseases in rheumatic diseases versus controls instead of raw numbers required for analysis, or included symptoms reflecting more than one disease. Among the few articles reporting raw data, several had incomplete data collection and contained errors (i.e., the numbers in tables do not add up to the summary data or the numbers mentioned in the text). Therefore, studies from (in particular northern European) countries with fairly homogeneous populations and virtually complete national disease registries are overrepresented.

### 3.1 Linear Incidence of Autoimmune Arthritis

**FIGURE 1:**
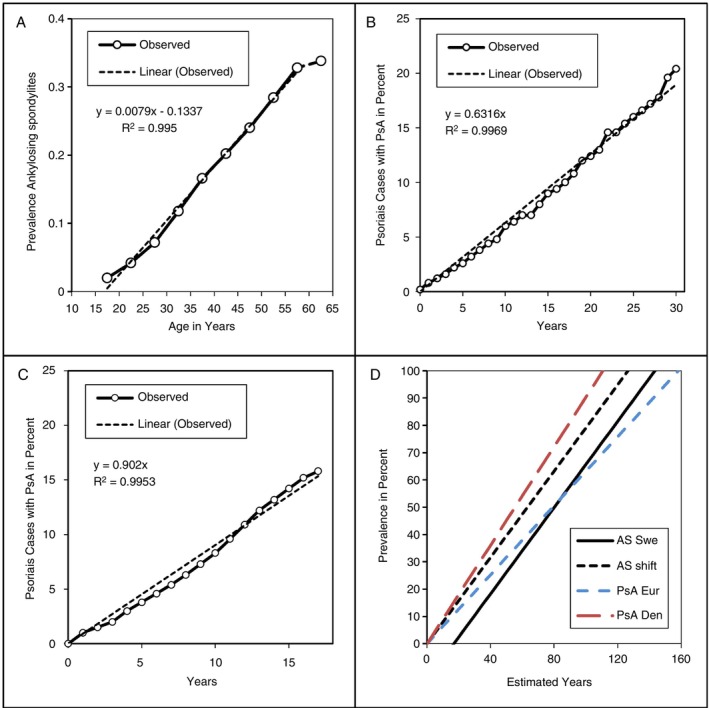
Linear prevalence of ankylosing spondylitis (AS) and psoriatic arthritis (PsA). (A) Age of diagnosis of AS in the population of Sweden. There is a shift in disease recognition of about 17 years, assuming the risk is present at birth; this shift likely is a combination of the time between the onset of disease and the onset of symptoms and the time between the onset of symptoms and diagnosis of AS. (B) Interval in years between first diagnosis of psoriasis in five countries in Europe—the United Kingdom, Italy, France, Spain and Germany—and the onset of PsA. (C) The same analysis as in (B) for all of Denmark. (D) Assuming a constant incidence of AS and PsA, all at‐risk people will have the disease after a hypothetical 110–160 years of follow‐up. Den—Denmark; Eur—Europe; shift—Swedish data shifted about 17 years to cross the origin; Swe—Sweden.

If the rate of mutations is constant and a single mutation invokes a change in phenotype, then the incidence of rheumatic diseases among those at risk should be constant. Therefore, the prevalence increases linearly over time. This was tested for diseases with a very characteristic set of clinical features virtually assuring the correct diagnosis was made: people who developed ankylosing spondylitis and people with psoriasis who were carefully followed for the development of psoriatic arthritis. Figure 1A shows the prevalence for all persons with ankylosing spondylitis in the Swedish National Patient Register as reported in 2015 [10]. Autoimmune diseases with a long lead time, that is, the time between somatic mutation and display of phenotype, will be detected late in life. Thus, detection of ankylosing spondylitis starts late in childhood or early adolescence; the incidence declines after 60 due to ageing of the immune system [11]. People from multiple locations in Europe with psoriasis were carefully monitored for the development of psoriatic arthritis, and the interval between the onset of psoriasis and psoriatic arthritis was measured in a study from 2010 [12]. Over a time span of 30 years, a perfect cumulative incidence was observed (Figure 1B). A similar 2018 study determined over an 18‐year period the interval between first‐time diagnosis of psoriasis in the entire population of Denmark and the development of psoriatic arthritis; again, a constant incidence was observed (Figure 1C) [13]. Therefore, both rheumatic diseases showed the expected constant incidence and linear prevalence. The *R* ^2^ for linear regression for all three studies was > 0.99. The rate of disease development depends on the nature of the gene pool and complete data collection; therefore, rates are different for each study (Figure 1D). Moreover, under the assumptions of a constant increase and a theoretical life expectancy of 160 years, it will take between 110 and 160 years for every at‐risk person to develop the disease. This is compatible with a dominant inheritance with incomplete penetrance as predicted by the new theory.

### 3.2 Distribution of AAIDs in Rheumatic Diseases

**FIGURE 2:**
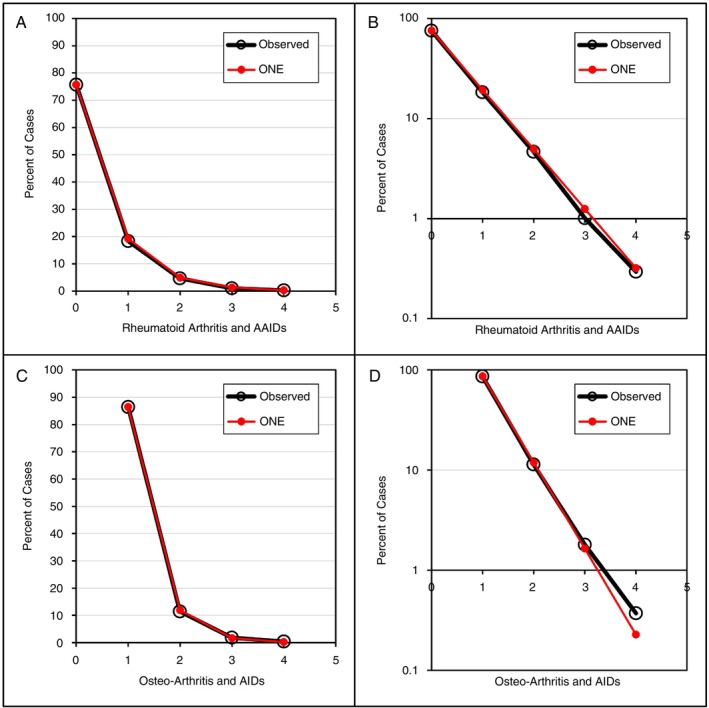
Distribution of AAIDs and its scalability to > 1 million persons. (A–D) Number of AAIDs or autoimmune diseases (AIDs) is shown on the *X*‐axis. The percentage of cases with one or more AAIDs or AIDs is shown on the *Y*‐axis. (B, D) The log_10_ transformation of ONE is shown on the *Y*‐axis. (A, B) Rheumatoid arthritis. (C, D) Osteo‐arthritis. Because osteo‐arthritis is not an AID, cases without AIDs cannot be included in the analysis.

A US study from 2017 reported the presence of one or more AAIDs in 286 601 persons with RA with an average age of 53.2 years [14]. Figure 2A,B shows that about 24.2% had one or more AAIDs; the observed distribution was perfectly modelled by ONE (2). The PCC was 1. The presence of autoimmune diseases was also studied in 1 421 624 persons with osteoarthritis (OA); the average age was 54 years. Only 10.5% had one or more autoimmune diseases, yet Figure 2C,D shows that the distribution of one or more autoimmune diseases was accurately modelled by ONE (2); the PCC was 1. The following AAIDs were reported in decreasing frequency (RA from 3.78% to 0.05%; OA from 0.69% to 0.01%): lupus, psoriatic arthritis, Sjogren's/sicca syndrome, psoriasis only, T1D, Interstitial lung disease/pulmonary fibrosis, polymyalgia rheumatica, ankylosing spondylitis, Crohn's disease, Raynaud's syndrome, ulcerative colitis, chronic urticaria, pernicious anaemia, Hashimoto's thyroiditis/autoimmune thyroid disease, vasculitis, autoimmune disease not elsewhere classified, uveitis, systemic sclerosis/scleroderma, sarcoidosis, scleritis/episcleritis, multiple sclerosis, polymyositis, Graves' disease, Addison's disease, dermatomyositis, celiac disease, giant cell arteritis, thrombocytopenic purpura/immune thrombocytopenic purpura, morphoea, erythema nodosum, alopecia areata, haemolytic anaemia, chronic glomerulonephritis, myasthenia gravis, primary biliary cholangitis, vitiligo and leukocytoclastic vasculitis.

**FIGURE 3:**
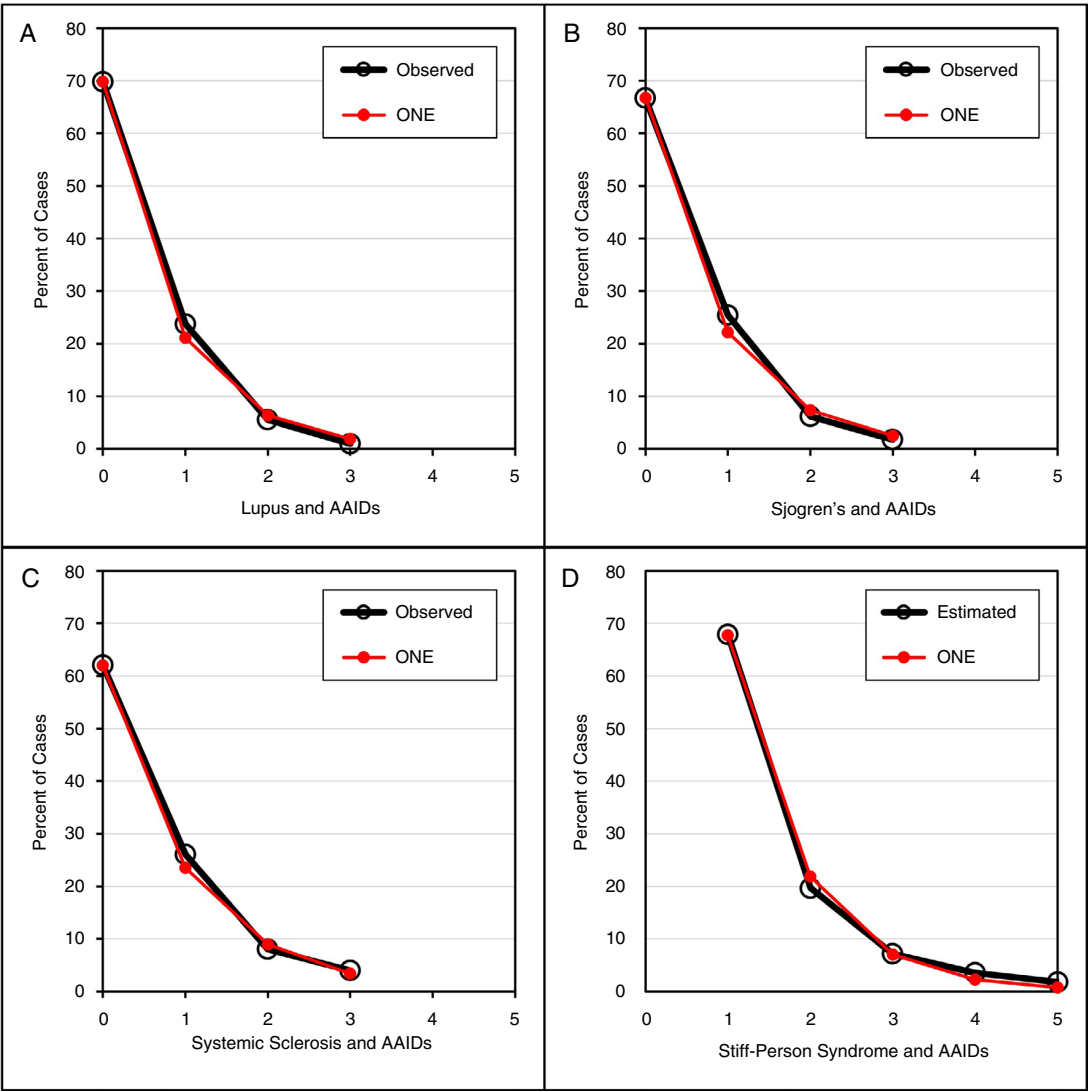
Distribution of AAIDs. (A–C) The primary autoimmune disease under study and AAIDs are shown on the *X*‐axis; the percentage of cases with one or more AAIDs is shown on the *Y*‐axis. (D) For SPS, only AAIDs are shown as there are two conditions with anti‐GAD antibodies (see Section 4). The result is an overlap syndrome where two autoimmune diseases are present in many cases (SPS and T1D); therefore, the assumption that a single mutation is the cause of SPS and AAIDs does not apply to all cases but only to AAIDs.

Next, a United Kingdom study from 2000 analysed 294 persons with lupus; one or more AAIDs were present in 30.2% [15]. The AAIDs included Sjögren's syndrome, RA, autoimmune thrombocytopenia, anti‐phospholipid syndrome, hypothyroidism, polymyositis, hyperthyroidism, myasthenia gravis, celiac disease, fibrosing alveolitis, juvenile chronic arthritis and chronic active hepatitis. The observed distribution, as shown in Figure 3A, was nearly perfectly modelled by ONE (2); the PCC was 0.999.

Among 114 persons with Sjogren's disease from England, 33.3% were found to have one or more AAIDs as reported in 2005 [16]. The AAIDs included hypothyroidism, Grave's disease/thyrotoxicosis, scleroderma, pulmonary fibrosis, chronic active hepatitis, primary biliary cholangitis, discoid lupus, myositis, renal tubular acidosis, glomerulonephritis, idiopathic thrombocytopenia, celiac disease and pernicious anaemia. Figure 3B shows a close correlation between observed and modelled data; the PPC was 0.998.

The presence of AAIDs in systemic sclerosis was studied in 2017 among 50 persons in Israel [17]. AAIDs included lupus, RA, myositis, ulcerative colitis, anti‐phospholipid syndrome, Sjogren's syndrome, vasculitis, polychondritis, autoimmune thyroid disease, sarcoidosis and primary biliary cholangitis. The correlation between observed and modelled data was excellent (Figure 3C); the PPC was 0.999.

The last disease investigated was SPS. A US study from 2012 reported that 56 among 99 persons with this disease had a total of 85 AAIDs [18]. The AAIDs consisted of T1D in 36 (43%) persons, thyroid disease in 28 (33%), pernicious anaemia in 7 (8%), vitiligo in 7 (8%) and ovarian failure, Addison disease, Sjogren's syndrome, lupus, celiac disease and an unknown disorder each in 1 person (8%). A precise distribution was not listed, but the observed data fit an exponential distribution of 38 cases with one, 11 with two, 4 with three, 2 with four and 1 with five AAIDs, as shown in Figure 3D; the PCC was 0.999. The disease distribution results are also compatible with a dominant inheritance with incomplete penetrance.

### 3.3 Diversity of AAIDs in Rheumatic Diseases

For common rheumatic diseases, such as RA, lupus, Sjogren's disease, systemic sclerosis, and the rare disease SPS, the variety of AAIDs was wide. GWAS, when available, of these diseases all suggest a dominant etiologic role for the MHC genes as predicted by the new theory [2]. The distribution of AAIDs followed the expected exponential distribution and thereby suggests that AAIDs were related to DNA damage of the same gene involving the same MHC epitope‐binding groove locus but resulted in a different mutation of the epitope‐binding groove resulting in a different self‐peptide being recognised as non‐self. Depending on the most likely damage of the mutation‐prone DNA site and the repair options, in some autoimmune diseases, certain AAIDs are more likely than others; a good example is SPS, where T1D and thyroid disorders accounted for 75% of AAIDs. The opposite example is RA, where the most frequently observed AAID, lupus, accounted for 3.8% of AAIDs. These observations agree with the new theory.

### 3.4 Order of AAIDs in Rheumatic Diseases

**FIGURE 4:**
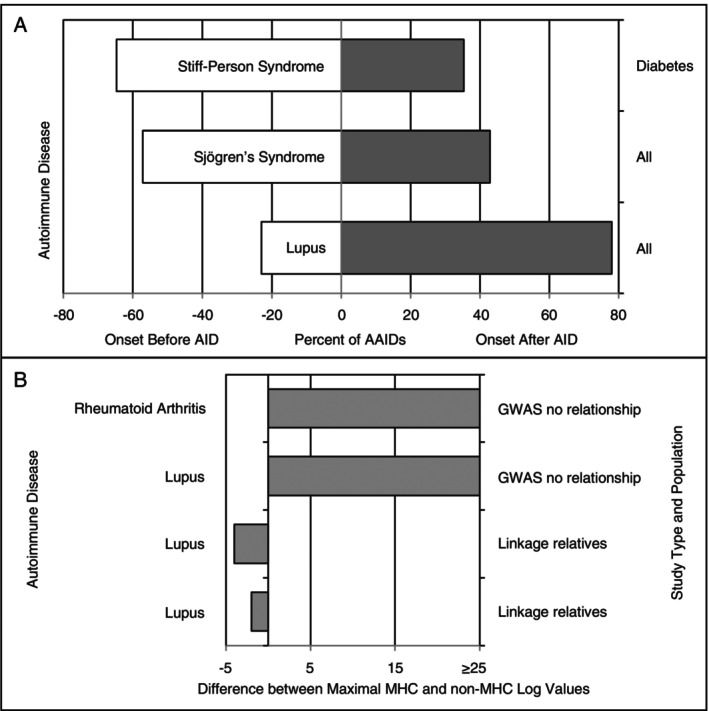
(A) The onset of AAIDs as available among articles; on the right *Y*‐axis, the AAIDs studied are shown. For SPS, only the onset of T1D was investigated; for Sjogren's syndrome and lupus, all AAIDs were included (AID: autoimmune disease being studied). AAIDs can occur before (white) or after (grey) the rheumatic disease. (B) GWAS of random populations with autoimmune rheumatic diseases consistently show maximal significance scores for MHC loci that greatly exceed non‐MHC loci (top two rows). This means that the MHC is the most important at‐risk factor for the rheumatic disease. However, in linkage studies of affected persons and relatives or heterozygous persons, the maximal significance scores for MHC loci decline, and non‐MHC loci will show greater significance (bottom two rows). Linkage cannot separate persons with the at‐risk gene with and without disease; hence, the MHC may appear less important than other genes, as shown here for lupus.

The order of autoimmune diseases within persons with more than one appears random. For instance, in the study of people with SPS, 22 persons acquired T1D 1–33 years before, and 14 T1D 1–12 years after the onset of SPS symptoms, which occurred at the median age of 40 years [18]. Figure 4A shows the percentage of AAIDs that occurred before or after the onset of Sjogren's syndrome, lupus and SPS [15, 16]. These observations agree with the new theory, which predicts that any autoimmune disease is the result of a random event that can occur before or after another autoimmune disease, given the random DNA damage and unpredictable repair sequence.

### 3.5 Linkage Studies vs. GWAS in Rheumatic Disease

Linkage studies assume that a specific DNA sequence is associated with a specific phenotype. For truly dominant and recessive genetic diseases, linkage studies are successful because the germline profile and phenotype match closely. As concluded above, autoimmune rheumatic diseases are caused by somatic mutations of at‐risk DNA with very low, incomplete penetrance. That implies the absence of a direct link between germline profile and phenotype; thus, linkage is unlikely to identify genes with incomplete expression and may exclude MHC genes [19]. On the other hand, GWAS of unrelated people will find DNA sequences that are more commonly present among people with the disease under study [20, 21]. Figures 1, 2, 3 show that about 20%–40% of the population with one autoimmune disease will experience one or more autoimmune diseases; by shifting the curve to the right and assigning the disease under study as the first autoimmune disease, the at‐risk population without any autoimmune disease (*X* = 0) can be estimated at 60%–80% of the total at‐risk population. Therefore, more people with the at‐risk MHC gene will not have disease, resulting in a false‐negative linkage for the MHC locus (Figure 4B) [19, 22].

### 3.6 Single or Multiple SHM Hotspots

The presence of autoimmune rheumatic disease depends on at‐risk DNA and age of the person, as shown in Figure 1. Assuming a dominant trait with a 30%–35% overall ‘at‐risk family penetration’ rate (the average penetration rate for all persons in a pedigree with an average age of 40 years), half of the family members will harbour the trait and one‐third of those will have developed disease. Therefore, one can expect to see one or more rheumatic and other autoimmune diseases in about 0.5 × 32.5% or 16% or one in six persons in families with autoimmune rheumatic diseases. While germline sequences associated with MHC‐based rheumatic diseases are known, for example, the ‘shared epitope’ sequences in HLA‐DRB1*04:01, 04:04, 04:05 predispose to RA and are enriched in SHM hotspots, the problem is that the sequences of at‐risk MHC DNA after somatic mutation are not known [2]. However, wild‐type and germline mutation sequences are known in monogenic, non‐MHC arthritis with incomplete penetrance and known point mutations; therefore, these rheumatic diseases may provide an opportunity to test the validity of the somatic mutation concept.

**FIGURE 5:**
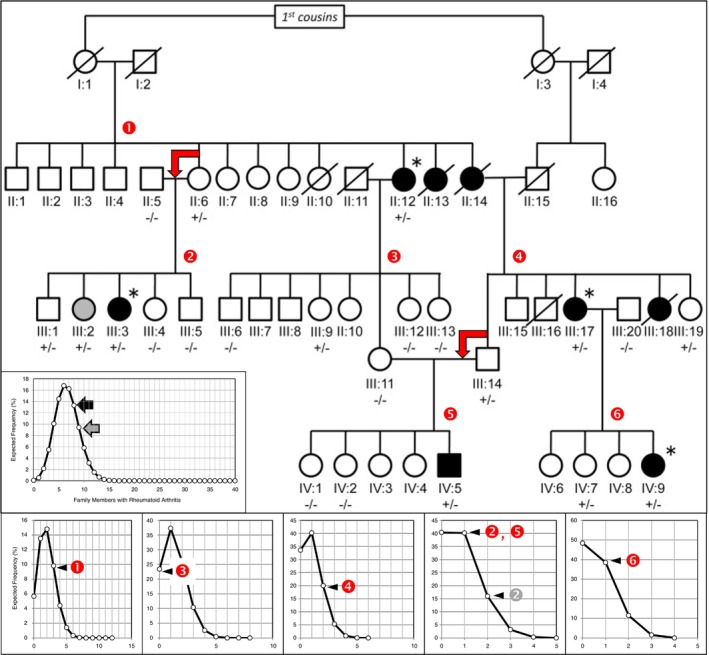
Pedigree of a family with PLB1 rheumatoid arthritis (RA) demonstrating incomplete penetrance, assuming an overall family chance to develop RA of 33.3%, that is, *X* = 0.333. In generations II, III and IV, there are 12, 19 and 9 members, respectively, at risk for having the disease trait: the allele that may cause RA. There are two instances of generation skips: Couples without RA having children with RA (red angled arrows); on average, transmission of the RA trait will happen in half of the children where one parent carries an RA allele but, assuming a chance of 0.333, only about one‐third of the children with the RA trait will develop clinical RA. Among 40 siblings (Generation II: 1–4, 6–10 and 12–14; Generation III: 1–19; and Generation IV: 1–9), between 4 and 10 are predicted to have RA (large inset). The observed number of eight members with clinical RA (black circles and square) and one member with antibodies typically seen in people at high risk for future RA (III: 2; grey circle) are within expected random variation of gene inheritance as shown in the large insert on the lower left (black arrow indicates the chance that eight members have clinical RA; grey arrow includes the member with antibodies). The small inserts at the bottom show for each couple (the numbers in red circles refer to the matching number in the pedigree; the number in grey includes for family 2 the member with antibodies) at risk for offspring with RA, the expected frequency of having one or more children with RA and the actual number. Figure modified from Okada et al. [23].

**FIGURE 6:**
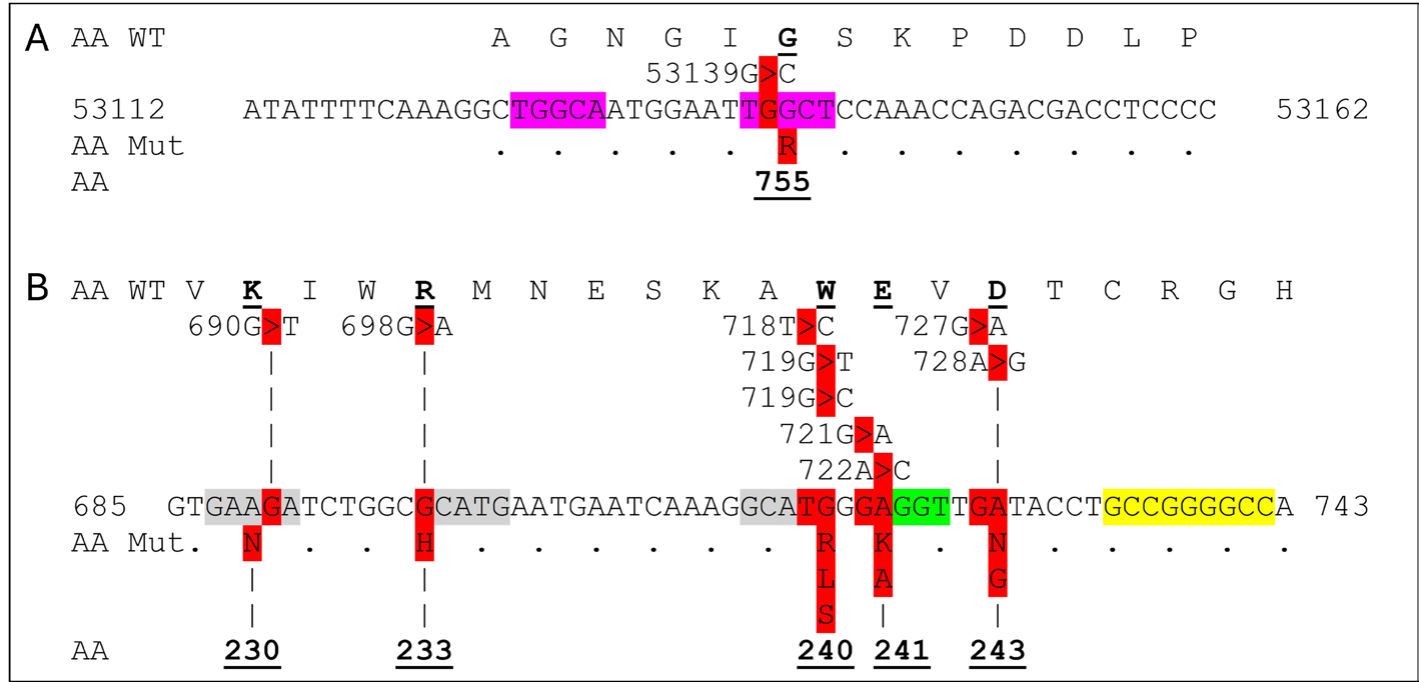
The sequence of germline mutations for two monogenic rheumatic diseases. (A) The start of exon 33 of the PLB1 gene contains two SHM hotspots (purple sequence). People at risk for PLB1 arthritis have a mutation in the second SHM hotspot, 53139G>C, which causes a G755R change (red sequence) in the CDS and protein. The new theory predicts that a mutation of the wild‐type allele will occur once every 100–150 patient years and create biallelic mutations at codon 755, creating a cell without a normal functioning PLB1 protein. The mutated locus—TCGCT—is not prone to additional mutation, but the wild‐type allele is part of a hypermutation hotspot (RGYW: TGGCT) that may generate a cysteine, arginine or stop codon [24]. (B) The end of exon 8 and start of exon 9 of the COPA CDS contain four SHM hotspots (grey and green sequences). Any of the mutations shown in the COPA gene is associated with an inflammatory syndrome that includes arthritis. The new theory predicts that a mutation in any of the four SHM hotspots—at nucleotides 690, 698, 718, 719, 721 and 722—of the wild‐type allele will occur at least once every 14–25 years, resulting in a cell with biallelic loss of normal COPA protein and an inflammatory syndrome with a very high disease penetrance starting in childhood. (A, B) SHM hotspots are shown in purple, green and grey colours; each hotspot is five nucleotides in length. Purple: Exact RGYW. Green: One mismatch RGYW. Grey: Exact amino acids (AA) or Other [2, 24]. A GC‐rich sequence of at least nine consecutive nucleotides is shown in yellow. DNA mutations are shown in red above the sequence, and AA mutations in red below the sequence. Bold, underlined text: Amino acid and position in CDS reported to cause PLB1 arthritis or COPA when mutated. Mut, mutation; WT, wild‐type. Red ‘>’ indicates mutation and shows the nucleotide position of the DNA mutation; the three digits of the amino acid position match the three nucleotides defining the AA or are tagged by vertical bars.

A pedigree of a large family with non‐MHC‐based, monogenic RA illustrates all aspects of the inheritance of MHC‐based autoimmune diseases: the presence of disease in one out of six persons and generation skips (Figure 5) [23]. Only a single autosomal mutation is associated with disease (PLB1 +/−). For each couple (small insets) and the entire pedigree (large inset), the likelihood was calculated that offspring have arthritis; the results show that the observed distribution of arthritis among family members is among the likely distributions predicted by the new theory. From this observation, multiple conclusions can be drawn. First, because people in this pedigree are not born with arthritis and the inheritance is dominant with incomplete penetrance, a single germline mutation on one allele is not sufficient to cause disease. Yet, the trait is passed to the next generation despite the absence of disease (red angled arrows). Second, the rate of somatic mutation is between once every 100–150 years, similar to the rate of MHC‐based rheumatic diseases (Figure 1D). In this pedigree, 8 out of 40 members (~20%) have arthritis. Because MHC‐based mutations involve SHM hotspots, the rate of arthritis penetrance suggests that mutation of the second allele occurs in or around a SHM hotspot. Third, the disease is extremely rare; the chance that two mutations occur in the same cell, each on a different allele is extremely small; if we assume 1 million cells are able to cause the disease through clonal expansion, and one critical mutation every 100 years per PLB1 allele in any cell, then the chance of two critically mutated alleles—one in each of the two alleles—at the same locus in the same cell is once every 10^6^ × 100^2^ or 10^10^ persons, or less than once among all living people. Therefore, the second mutation likely occurs in the same locus as the first mutation; if there is more than one SHM hotspot able to cause disease, then the rate of disease would be greater than one in six, and the disease would not be as rare as reported. Finally, the second somatic mutation must cause a biallelic PLB1 protein loss‐of‐function in the affected cell; this must start an activation cascade resulting in inflammation that starts the arthritis phenotype [5]. In this family, the dominant inheritance with incomplete penetrance was caused by a single germline mutation on one allele of the PLB1 gene. Figure 6A shows the genomic sequence of the PLB1 gene surrounding the start of exon 33. The predictions of the new theory related to DNA are valid: the inherited mutated allele originated in a SHM hotspot (second purple sequence), which is present on the second, normal allele. The inherited mutated locus has ‘probably damaging’ repercussions according to PolyPhen‐2.0 analysis [23]. Deletion of parts of exons 5 and 6 with flanking intronic sequence in a mouse model is predicted to result in early truncation; this does not appear to cause an arthritis phenotype [25].

If the interpretation of the pedigree for mutated PLB1 and a double mutation in a single cell, followed by an inflammation cascade, is the cause of PLB1 arthritis, then a gene with multiple SHM hotspots, each causing disease through somatic mutation in the presence of one germline mutation on the complementary allele, should have nearly complete penetrance at an early age. COPA syndrome is a recently described disease associated with heterozygous germline missense mutations in coatomer protein complex subunit α (COPA) that has a ~75% penetrance rate and causes an inflammatory syndrome with lung, kidney and joint involvement most of the time in childhood [26]. By 2021, 10 germline mutations associated with COPA syndrome were reported within a 160‐nucleotide CDS sequence coding a WD40 motif; 9 of the 10 are in the first 60 nucleotides (Figure 6B). The wild‐type 160 nucleotide sequence contains seven SHM hotspots and a GC‐rich DNA sequence; 7 of the 10 mutations are located within the first four SHM hotspots (grey and green sequences) [24]. Any missense somatic mutation within the 160 nucleotide sequence of the wild‐type allele likely results in two abnormal COPA proteins; with four SHM hotspots and seven known mutations among these hotspots, the chance of this happening in a cell, assuming a rate of one mutation every 100 years, is expected to occur about every 100/4 to 100/7 years or every 14–25 years. This explains the early onset of COPA and high penetrance rate. Two mutant proteins also explain the phenotype as many mice heterozygous for a human Gly241Lys mutation knock‐in develop in time lung disease similar to the lung disease observed in people, whereas mice with homozygous germline COPA mutations die in embryo [27].

## 4 Discussion

The linear prevalence, distribution, order of development and diversity of AAIDs in rheumatic diseases support the concept that all DNA is damaged at a constant rate and, as a result, mutates at a constant rate [1]. A constant rate of damage should fit a simple, linear model. Indeed, the ONE model is simple and fits the observed data convincingly; the PCC measures the strength of a simple, linear relationship and was > 0.99 or 1 for all results. If all DNA undergoes a constant rate of damage, then confounders such as sex, ethnicity, environment and diagnostic drift do not really matter because they do not affect the physical DNA damage. In fact, the model performed equally well in populations in Europe, the Middle East, Asia and the Americas. The new theory appears robust, but sensitivity analyses could not be conducted for disease distribution because none of the rheumatic disease datasets identified contained sufficiently detailed numeric data to allow this. However, these analyses were possible for T1D (with or without thyroid disease) and for head and neck cancers (with lung, oesophageal and bladder cancers); all sensitivity analyses were published in 2024 and 2025, and the PCCs for the analyses were 0.999–1 [2, 3]. A variant of sensitivity analysis is the analysis shown in Figure 2; here, the primary disease under study, RA, was removed and replaced by OA. The distribution of one or more autoimmune diseases among people with this non‐autoimmune condition adhered tightly to ONE (2); the PPC was 1.

If damage to multiple DNA loci on the same gene can initiate disease, then disease prevalence increases with onset at an earlier age. Not all loci within DNA mutate at the same rate; SHM hotspots and GC‐rich DNA are mutation‐prone and more frequently sites of mutation [2, 6]. Yet, even in mutation‐prone DNA, the rate of somatic mutations is low and therefore not all people with at‐risk DNA develop rheumatic diseases; this explains the incomplete penetrance of about 30%–35% in 40‐year‐old at‐risk people. By combining three reports containing patient cohorts with different average ages from northern Italy, the risk of celiac disease as primary or AAID in primary biliary cholangitis and AAIDs was assessed [28]. The prevalence of autoimmune diseases was linear, and the risk for any autoimmune disease was about 31% at age 40 or once per 120 patient years, similar for autoimmune rheumatic diseases, as shown in Figure 1D. A damage‐based aetiology also explains why there is no order among autoimmune diseases in people with more than one autoimmune disease, as shown in Figure 4A. Therefore, suggestions that a specific autoimmune disease predisposes one to another specific autoimmune disease are invalid; the correct explanation is that, given the underlying DNA sequence, certain autoimmune diseases are more likely to occur together. Dominant inheritance with incomplete penetrance explains why simple linkage analysis among family members with rheumatic diseases may exclude causative genes such as HLA genes in the MHC region (Figure 4B).

Both PLB1 arthritis and COPA syndrome are classified as being inherited in a dominant fashion. The variable age of onset and the incomplete penetrance, however, suggest that specific events need to occur that initiate the disease. A growing number of non‐malignant diseases appear to start when both alleles in a cell are inactivated or produce dysfunctional proteins: biallelic loss‐of‐function [5]. An example is porokeratosis, a mevalonate kinase‐associated disease. In porokeratosis multiple, localised skin lesions develop due to a mevalonate‐kinase mutation; the skin lesions are characterised by tightly packed parakeratotic cells surrounded by atrophy and inflammation. Similar to polyps, the skin is easily biopsied; genetic analysis has shown different mutational patterns among porokeratosis skin lesions [29]. All have the same germline mutation, but the second allele contains somatic mutations creating cells without normal protein compatible with the two‐hit hypothesis [5]. To avoid confusion with cancer, the terms ‘pseudo‐dominant’ inheritance with incomplete penetrance and somatic recessive expression due to the late occurrence of a second post‐zygotic loss‐of‐function mutation have been used [29, 30].

The frequency of AAIDs among many of the autoimmune rheumatic diseases is remarkably similar. When comparing the curves for RA, lupus, scleroderma and Sjogren's syndrome, the risk for AAIDs is between 25% and 40%. This suggests that somatic mutations causing different autoimmune diseases occur in similar at‐risk loci, which is compatible with the new theory: most common autoimmune (rheumatic) diseases are caused by somatic mutations in the epitope‐binding groove of HLA proteins [2]. However, the frequency of specific autoimmune diseases is determined by the likelihood that the repaired HLA gene results in a mutated HLA protein that binds a specific self‐antigen and starts an autoimmune cascade that results in a specific autoimmune disease.

An important outlier is SPS, which has an average age of 45, a risk for AAIDs of about 56% with the majority being T1D and vitiligo [18]. SPS and T1D are both associated with the presence of anti‐glutamic acid decarboxylase (GAD) antibodies. GAD is required for the synthesis of gamma‐aminobutyric acid (GABA), the major inhibitory neurotransmitter in the central nervous system that plays a critical role in both neurological function and insulin secretion by the pancreas [31]. Anti‐GAD antibodies may cause GABA insufficiency and thereby impair neurological function and insulin secretion, explaining why SPS is an outlier when it comes to the frequency of AAIDs.

DNA damage as the underlying cause of mutations may make more sense than at first sight. First, it unties mutations from cell division. Second, it unties mutations from cold or heat. Third, it associates gene transcription with somatic mutations. Fourth, it explains cluster mutations. And fifth, it explains karyotype changes as seen in pregnancy loss. In short, DNA damage explains all forms of DNA mutations, deletions and insertions as well as chromosomal translocations, rearrangements, deletions and duplications. DNA damage due to ionising radiation as proposed by the new theory also implies the possibility of weak links in the nucleotide chain of DNA. In theory, weak links can occur at the 3D structure or at specific DNA sequence motifs. Obviously, single‐strand unwound, unprotected DNA during transcription or DNA crossovers between homologous chromosomes during M phase of cell division creates 3D structures more easily physically disrupted than double‐strand helix DNA wound around histones. Single‐cell sequencing of neurons confirms this prediction of the new theory: somatic mutation in single human neurons tracks developmental and transcriptional history [32]. The sensitivity of DNA in an unprotected state to physical damage leading to single or clustered lesions is used in radiation oncology to treat cancer, especially cancers with a rapid rate of cell division [33].

The new theory prompted an investigation into possible weak links due to specific sequence motifs. Accepted mutation‐prone motifs include microsatellites and CpG‐rich sequences. These motifs typically do not cause point mutations in exons and thereby do not cause specific protein dysfunction. Yet the exponential tapering distribution of multiple events, that is, AAIDs in rheumatic diseases, suggests a defined, limited, single somatic mutation. The best‐known mechanism of purposeful DNA rearrangement and creation of point mutations in a controlled fashion is the process of antibody selection and diversification. The smallest changes during antibody diversification are created through mutations in SHM hotspots [24, 34]. Therefore, it was hypothesised that the mutation‐prone nature of SHM hotspot DNA sequences is a general feature, although the mutability in SHM hotspots not associated with the antibody diversification process is reduced. The hypothesis was found to be true for sites of frequent germline mutation in the APC gene (adenomatous polyposis coli in colorectal cancer), the APP gene (beta‐amyloid in Alzheimer's disease) and the SNCA gene (synuclein‐alpha in Parkinson's disease) [6, 35]. Moreover, in colorectal cancer, somatic mutations preferentially occur in SHM hotspots [6]. For T1D, the greatest diversity in sequence and presence of SHM hotspots was found in sequence defining the P4 pocket of the DRB1*04 epitope‐binding groove [2]. DRB1*04 is also strongly associated with RA, lupus, systemic sclerosis and mixed connective tissue disease. In this study, it was shown for PLB1 arthritis and COPA syndrome that the number of SHM hotspots determines the rate of somatic mutations and thereby explains the average age of disease onset and the degree of disease penetrance.

The new theory poses that an important source of DNA damage is minimum ionising particle (MIP) radiation. MIP radiation as a major source of DNA mutations has been contemplated during the past century, but the lack of evidence led to the current opinion that it is not important and should not be considered [3, 36, 37]. Yet, recent findings indicate that we need to reconsider a possible role of MIP radiation [1, 3, 38]. All life at Earth's surface level is continuously exposed to MIP radiation, which consists mainly of muons, a short‐lived, negatively charged, spinning particle with a mass 207 times that of an electron [1]. Muons emerge when galactic cosmic rays collide with nitrogen and oxygen molecules in the atmosphere 15–20 km above Earth's surface level. Muons hold on average an energy of 4 GeV; they are considered low linear energy transfer (LET) radiation, allowing most to travel through tissues without interacting extensively with atoms. Although about 1% of muon energy is absorbed by humans—energy proportionally decreases with mass traversed—and muons are sparse—1 muon per cm^2^ per minute—the amount of energy from a molecular standpoint is staggering: the average muon holds the energy to potentially cause 400 million double‐stranded or 1 billion single‐stranded DNA breaks. Or the formation of radicals, specifically 13.3 billion of the most common radical superoxide anions (O2^•−^), or about 1.3 billion hydroxyl (OH^•^) radicals. Muon effects include direct effects (collisions with or without particle shower formation), attenuation (knock‐off of electrons, oxidative stress, bremsstrahlung) and decay (into an electron and two types of neutrinos) with an energy range from a few eV to 4 GeV that is evenly distributed throughout the human body. Particle‐based damage also explains DNA cluster mutations and karyotype changes, for which currently there is no explanation. The spin‐polarised radiation of muons may be the cause of the right‐handed twist of the double DNA helix [39, 40]. Near absence of muons deep underwater or underground is associated with reduced or near absence of mutations [1, 41, 42, 43]. A recent review states that it is time to study the effects of muons in more detail in those locations [44]. Thus, muons can explain the right‐handed twist of DNA and a significant part of the mutation rate of all organisms at sea level. No other type of ionising radiation, whether ionising particles, x‐rays or gamma rays, approaches this feature set.

The new theory radically breaks with the current concept; it puts forward a single mechanism driving in large part the continued evolution of life on Earth, the development of intrinsic diseases, and the finality of life as it exists. That precludes a direct comparison with current disease concepts; those tend to be grounded in the belief of multiple different causes, each able to cause a disease. Moreover, the cause of most diseases is thought to be disease‐specific. Under the new theory, risk factors increase the chance of mutations or amplify the effects of mutations. Risk factors, therefore, are for the greater part not disease‐specific; hence, the concept of body‐wide field effects [3]. There is common ground in the modulation of risk; both current concepts and the new theory have similar risk factors, but in the new theory, all risk is directly related to the chance of DNA damage. Therefore, an unwound 3D DNA structure, weak DNA sequences, alterations to DNA via adducts, methylation changes or enzymatic activity and reduced ability to repair DNA are the main risk factors in the proposed theory.

In the new theory, biology is part of and directly influenced by the cosmos: the well‐ordered system that forms the universe [1]. If biology adheres to the Standard Model of particle physics, then biology measurements reflecting particle physics, that is, DNA damage, should be very consistent and barely fluctuate from predictions based on muon flux [45]. However, the highly accurate predictions in particle physics are based on billions of measurements—the latest G‐2 muon experiment collected data on 308 billion muons—under experimental conditions that are not possible for measurements of human diseases [46]. Yet, the agreement between observed data and ONE (2) based predictions is extremely accurate (frequently PCC > 0.9995), as would be expected if biology is governed by the Standard Model. Among all forms of radiation, only x‐rays and muons are true low LET radiation to which humans are regularly exposed [33]. Low LET radiation can deeply penetrate tissues and transfer energy throughout the entire body. As the muon flux shows only minor annual fluctuations, the risk of DNA damage linearly increases with muon dose [28]. Yet, although 5 mGy x‐rays are causally associated with cancer, an equal dose of muons is not [36, 37, 47].

This study has limitations. Study selection was stringent and focused on carefully followed‐up populations with reporting of raw data; thereby confounding factors were avoided. Linear prevalence and exponential distribution of immune events are compatible with a constant rate of DNA damage by ionising radiation, but there is no direct evidence for this in humans. Indeed, a constant rate of muons causes a linear rate of mutations in plants, for example, Tradescantia as shown in 1974, but this will be difficult to prove for human diseases [48]. Muons are the most common secondary cosmic ray particles at Earth's surface and the most likely source of DNA damage, but other secondary particles like electrons, positrons and neutrons, as well as terrestrial radiation such as Radon in air, Uranium and Thorium in soil and Potassium‐40 in our body, also contribute to DNA damage [1]. Fish such as rockfish, coelacanths and Greenland sharks, live most of their lifetime deep underwater in ice‐cold water and are known to live exceptionally long [49]. The current explanation for their longevity is slow metabolism and lower levels of oxidative stress due to the cold environment. This appears to make sense; however, the species with the lowest rate of mutation is a bacterial strain that lives 2–3 km underground at a temperature of 50°C–60°C; over a period of around 100 million years, it acquired about 30 point mutations [43]. Only the absence of muons explains the absence of mutations in hot and cold environments. There is also no direct evidence that proves the mutation‐prone nature of SHM hotspots outside the process of immunoglobulin diversification. That too makes sense at first sight. If several critical codons of key regulatory proteins are encoded by SHM hotspots, then survival would be affected because severe disease is predicted to develop in many at an early onset as in COPA syndrome. Only one of eight well‐known P53 mutations and none of the five KRAS mutations are encoded by SHM hotspots (unpublished observations). Indeed, germline missense mutations in HRAS and KRAS and in genes of molecules that function up‐ or downstream of HRAS and KRAS in cellular signalling networks cause related developmental disorders such as Costello syndrome, Noonan syndrome and cardiofaciocutaneous syndrome that limit offspring [50, 51]. However, in common chronic diseases where the malfunction of a protein in a single cell does not affect the generation of offspring, the presence of a germline mutation followed by somatic mutations in SHM hotspots on the second allele in many cells over decades will be associated with earlier onset and increased prevalence of disease in conditions such as FAP and Alzheimer's and Parkinson's disease [6, 52]. Thus, the importance of SHM hotspots appears protein‐specific.

In summary, a new theory explains the cause of autoimmune and non‐HLA, monogenic rheumatic diseases. A constant rate of MIP radiation, consisting predominantly of muons, causes a very low constant rate of DNA damage in all human tissues, of which most can be repaired. A very low rate of somatic point or small cluster mutations creates abnormal proteins, and when the mutation affects a critical protein domain, it may initiate a new phenotype, that is, rheumatic disease. The theory predicts that mutations preferentially occur in mutation‐prone DNA such as SHM hotspots and GC‐rich DNA sequences. For autoimmune rheumatic diseases, this requires a somatic mutation of a single at‐risk HLA locus on one allele, and for monogenic non‐HLA rheumatic diseases, a germline mutation on one allele followed by a somatic mutation on the other allele. The clinical presentation will be a dominant or pseudo‐dominant inherited rheumatic disease with incomplete penetrance. A greater number of SHM hotspots encoding critical amino acids associated with monogenic non‐HLA rheumatic diseases explains the time of onset of disease and rate of disease penetrance. Additional typical features of rheumatic diseases are also explained; these include random occurrence of AAIDs before or after a rheumatic disease, the exponential distribution of AAIDs and the failure of simple linkage to find causal genes when somatic mutations contribute to biallelic loss‐of‐function.

## Funding

This work was supported by the National Institute of Diabetes and Digestive and Kidney Diseases (DK106130) and the University of Minnesota.

## Conflicts of Interest

The author declares no conflicts of interest.

## Data Availability

The data that support the findings of this study were retrieved from peer‐reviewed articles on the internet in public and publisher domains; articles were identified via queries in PubMed at https://pubmed.ncbi.nlm.nih.gov/.
